# Long COVID and symptom trajectory in a representative sample of Americans in the first year of the pandemic

**DOI:** 10.1038/s41598-022-15727-0

**Published:** 2022-07-08

**Authors:** Qiao Wu, Jennifer A. Ailshire, Eileen M. Crimmins

**Affiliations:** 1 MIPM, Leonard Davis School of Gerontology, Andrus Gerontology Center, University of Southern California, 3715 McClintock Ave, Room 215, Los Angeles, CA 90089-019 USA; 2 Leonard Davis School of Gerontology, University of Southern California, Los Angeles, CA USA

**Keywords:** Diseases, Risk factors

## Abstract

People who have COVID-19 can experience symptoms for months. Studies on long COVID in the population lack representative samples and longitudinal data focusing on new-onset symptoms occurring with COVID while accounting for pre-infection symptoms. We use a sample representing the U.S. community population from the Understanding America Study COVID-19 Survey, which surveyed around 8000 respondents bi-weekly from March 2020 to March 2021. Our final sample includes 308 infected individuals who were interviewed one month before, around the time of, and 12 weeks after infection. About 23% of the sample experienced new-onset symptoms during infection which lasted for more than 12 weeks, and thus can be considered as having long COVID. The most common new-onset persistent symptoms among those included in the study were headache (22%), runny or stuffy nose (19%), abdominal discomfort (18%), fatigue (17%), and diarrhea (13%). Long COVID was more likely among obese individuals (OR = 5.44, 95% CI 2.12–13.96) and those who experienced hair loss (OR = 6.94, 95% CI 1.03–46.92), headache (OR = 3.37, 95% CI 1.18–9.60), and sore throat (OR = 3.56, 95% CI 1.21–10.46) during infection. There was a lack of evidence relating risk to age, gender, race/ethnicity, education, current smoking status, or comorbid chronic conditions. This work provides national estimates of long COVID in a representative sample after accounting for pre-infection symptoms.

## Introduction

While SARS-CoV-2 is usually thought of as an acute disease, we now know that some people with COVID experience a variety of post-acute health problems long after their disease onset. This experience of long-term persistent symptoms has been termed “long COVID”^1,2^. Acute COVID typically lasts 3 weeks^2–4^, but long COVID can last weeks, months, or longer^5^. Studies have reported widely varying prevalence levels of long COVID, ranging from 10% to over 90%. However, a lack of consensus and the evolving diagnostic criteria for long COVID have resulted in inconsistent definitions of long COVID. At the same time, limitations in study design have made it difficult to obtain a valid estimate of the prevalence, symptoms of, and associated risk factors for long COVID in the United States population.

Early evidence of long COVID was based on discharged hospitalized COVID patients in a number of countries. Since these reflected patients with worse disease outcomes than average, the estimated prevalence of long COVID was generally high, ranging from 50 to 90%^6–11^. However, hospitalized patients account for a very small proportion (about 5%) of COVID-19 cases^12^; so, focusing only on samples of the discharged hospitalized patients provides a limited perspective on the experience of long COVID in the broader population. Studies using samples combining hospitalized and non-hospitalized individuals from specific geographic regions generally reported lower prevalence of long COVID compared to those focusing only on hospitalized patients, mostly ranging from 30 to 70%^1,13–20^. A recent meta-analysis of studies with both hospitalized and non-hospitalized individuals concluded that the prevalence is closer to 33%^21^. But the prevalence of long COVID is also highly dependent on the duration of follow-up. Another meta-analysis found prevalence of 63%, 72% and 46% at 30, 60, or 90 + days after onset or hospitalization with COVID^22^. Thus, estimated prevalence may be higher when the sample includes a greater proportion of hospitalized patients, who are likely to have had more severe infections, or shorter time frames in which post-infection symptoms are more likely to be observed.

Only two population representative studies on long COVID have been conducted to-date. In December 2020, the Office for National Statistics (ONS) estimated the prevalence of long COVID in the U.K. from a survey of 8,193 non-hospitalized and non-institutionalized respondents who ever tested positive for COVID during the survey follow-up—21% exhibited symptoms lasting longer than 5 weeks, and 10% exhibited symptoms lasting longer than 12 weeks^23^. In June 2021, using data from the Real-time Assessment of Community Transmission-2 (REACT-2) Study, a nationally representative sample of the community population in England, Whitaker et al.^24^ reported that among 28,713 respondents who reported a valid date of symptom onset, 38% experienced at least one symptom for more than 12 weeks. These two studies indicated lower prevalence of long COVID prevalence compared to other previously discussed studies; probably because they are more population representative, and include lower proportions of hospitalized individuals. Importantly, these estimates are based on respondents’ symptoms reported after infection but did not consider symptoms prior to the infection.

Long COVID can affect multiple organs and body systems and can be reflected in a wide range of long-lasting symptoms^4^. According to public health agencies^25,26^, the most commonly reported long COVID symptoms include respiratory abnormality, tiredness, neurocognitive problems, pain, flu-like symptoms, changes in smell or taste, as well as symptoms related to the cardiovascular system, digestive system, hair, and skin. Some symptoms of COVID are fairly common symptoms occurring also in non-COVID persons. Symptoms included in assessment of long COVID, such as fatigue, headache, body ache, sneezing, and dry skin, are common to other health conditions and may be linked to seasonal environmental conditions even among the healthy population. Sudre et al.^27^ conducted the only study we know of that accounted for pre-infection symptoms and found much lower long COVID prevalence, however this was a convenience sample of app users and therefore not population representative. The lack of longitudinal data prior to infection has thus far precluded a close examination of this. It has been acknowledged that not accounting for pre-infection symptoms may result in overestimation of long COVID prevalence^24,28^.

In addition to interest in the population prevalence of long COVID, it is important to determine if long COVID risk differs across the population. Previous studies have found older adults^23,28^ and women^17,24,27,28^ to have elevated risks of long COVID. Existing health conditions may increase the risk of long COVID as well. The most commonly and consistently reported long COVID risk factor is obesity^24,27,29^, which is also one of the strongest risk factors for severe COVID illness^30^. Pre-existing conditions in general^24,28,29^ and asthma^29^ are also found to be associated with higher risk of long COVID. Symptoms during acute COVID might predict long COVID as well. Augustin et al.^16^ found the people experiencing anosmia and diarrhea are more likely to develop persistent symptoms. According to the CDC, in the U.S., socioeconomic and environmental factors are associated with increased risk of exposure to COVID, and these factors disproportionately affect racial and ethnic minority groups^31^. Though there is no direct evidence suggesting that those factors are associated with the risk of long COVID, racial/ethnic minorities and people with low education may be more at risk for long COVID as well.

In the current study, we used survey data from a nationally representative sample of community-dwelling U.S. adults, conducted from March 2020 to March 2021 to (1) estimate the baseline-symptom-adjusted prevalence of long COVID, (2) show the most commonly reported long COVID symptoms, and (3) identify the risk factors of becoming a COVID long hauler.

## Methods

### Data

We used data from the Understanding America Study (UAS) COVID-19 National Sample, an ongoing longitudinal national probability-based internet panel of approximately 9,000 non-institutional U.S. adults administered by the Center for Economic and Social Research (CESR), at the University of Southern California (USC). The UAS uses two-stage stratified random sampling of individuals from households in U.S. zip codes. Respondents answer the survey using a computer, tablet, or smartphone and are provided a tablet and broadband internet if needed^32^. The UAS started administrating the longitudinal COVID-19 national survey to its panel members in March 2020^33^. Follow-up surveys were fielded every two weeks beginning April 1, 2020. Over each 2-week survey period, one-fourteenth of the respondent pool was asked each day to fill out the survey within 2 weeks. More than 90% of the responses were completed in two weeks for each wave, and most were completed on the day of assignment.

The current study used the first 25 waves of the survey, which were collected biweekly from March 10, 2020 to March 31, 2021. During this period, 8,425 respondents participated in the survey, and 872 people (~ 10% of the total participants) reported that they were diagnosed with, or tested positive for COVID. We limited the analytic sample to 310 respondents who had COVID during the study period who also had information on self-reported symptoms at three times: At 4 weeks before reporting a COVID diagnosis/positive test, at the time of the report of COVID, and 12 weeks after the report of COVID. After excluding 2 respondents with missing information on covariates, our final analytical sample consisted of 308 people. In other words, 564 were dropped due to missing data. We did not find evidence of major differences between our final sample and those we dropped (Supplementary Table S1). The only statistically significant difference is that those who had missing data are more likely to be non-Hispanic others (p = 0.031). So, we were able to examine long COVID among a sample representing those who got COVID.

### Measures

#### COVID infection

The COVID infected population was determined based on questions about both COVID tests and diagnosis. Participants were asked: “Have you been tested for coronavirus since the last time you took our coronavirus survey? If so, what was the result?” and “Whether or not you have had a coronavirus test, has a doctor or another healthcare professional diagnosed you as having or probably having the coronavirus since the last time you took our coronavirus survey?”. We considered a respondent as having COVID who either tested positive to SARS-CoV-2 or who was diagnosed with COVID by a healthcare professional. In the early period of the survey, tests were less available than in the later part and people largely learned of their COVID status from a healthcare professionals. But as more people became infected and could obtain tests, people were urged not to access medical care unless symptoms were severe but to instead isolate at home. Thus, in the later period of the survey knowledge of infection came from both tests and diagnosis. For these reasons, we consider COVID infections from both testing and medical diagnosis to get the full picture of population prevalence.

#### Self-reported symptoms

There is no official list of clinical symptoms defining long COVID. We examined the 18 self-reported symptoms included in the survey. At each wave, respondents were asked to report whether they had experienced the following symptoms in the past 7 days: (1) fever or chills, (2) runny or stuffy nose, (3) chest congestion, (4) cough, (5) sore throat, (6) sneezing, (7) muscle or body aches, (8) headaches, (9) fatigue or tiredness, (10) shortness of breath, (11) abdominal discomfort, (12) vomiting, (13) hair loss, (14) dry skin, (15) body temperature higher than 100.4 F or 38.0 C, (16) diarrhea, (17) lost sense of smell, (18) skin rash. The response options included “Yes”, “No”, and “Unsure”. We treated “Unsure” as not reporting the symptom by combining “Unsure” with “No”. A symptom count variable ranging from 0 to 18 was then generated for each respondent at each wave by adding up the number of symptoms reported at a specific wave.

#### Long COVID

We consider a respondent as having long COVID if they met all of the following conditions: (1) reported a symptom at the time of the reported infection; (2) *did not* report experiencing that symptom 4 weeks prior to the reported infection; and (3) *continued* to report experiencing that symptom *at* 12 weeks after the reported infection. This definition distinguishes symptoms most likely caused by COVID from symptoms the respondent was already experiencing prior to infection.

For the purpose of comparison with other studies, we also alternatively define long COVID without adjusting for pre-infection symptoms, by considering all infected respondents who reported any symptom 12 weeks after reporting infection as long haulers.

#### Existing health conditions

Because underlying medical conditions have been linked to elevated risk for severe illness from COVID^34^, we examine whether existing health conditions are associated with increased risk of long COVID. Participants were asked “Have you ever been told by a doctor, nurse, or other health professional that you have any of the following medical conditions?”: (1) diabetes, (2) cancer (other than skin cancer), (3) heart disease, (4) high blood pressure, (5) asthma, (6) chronic lung disease such as COPD or emphysema, (7) kidney disease, (8) autoimmune disorder such as rheumatoid arthritis or Crohn’s Disease, and (9) obesity. Each condition was treated as a binary variable in the analyses.

#### Other covariates

Other covariates included age, gender, race/ethnicity, education level, and current smoking status. Age was categorized into three groups: ages 18 to 49, ages 50 to 64, and ages 65 and above. Race/ethnic groups included non-Hispanic White, non-Hispanic Black, Hispanic, and others. Education was classified as high school or less, some college education without a bachelor’s degree and a bachelor’s degree or more.

### Statistical analysis

We treated the survey wave when the respondents reported that they tested positive for, or were diagnosed with, COVID as the time infection was reported. By design, the survey interval was 2 weeks. Since the questionnaires were sent to the respondents biweekly, and most of the responses were completed on the day of assignment, on average, a person would have been diagnosed or had a test one week before reporting. So, two waves before the reporting date was, on average, 3 weeks prior to the date of infection, and 6 waves after reported infection was, on average, 13 weeks (and at least 12 weeks) after infection.

We first summarized the sample characteristics at the time of reported infection and compared them to the profile of the COVID-infected population in the United States provided by the Centers for Disease Control^35^ in order to assess the generalizability of the survey results. We also compared the characteristics of the long COVID group to those who experienced COVID but not long COVID. We tested for differences using t-tests for continuous variables and Wald tests for categorical variables.

Next, we estimated the prevalence of long COVID using two methods: using all reported current symptoms, to compare with estimates from previous studies, and using new symptom onset accounting for pre-infection symptoms.

We subsequently used our estimates of long COVID prevalence from the UAS sample and information on hospitalization and long COVID among hospital cases from other sources to make a national estimate of long COVID. Information on hospitalization is not available in the UAS but since hospitalized COVID patients generally experience moderate or severe disease outcomes, it is reasonable to assume that they are more likely to have missing data in the UAS and to be excluded from our final sample. This would lead to an underestimate of the prevalence of long COVID. So, we further provided an adjusted prevalence of long COVID using **Eq. (****1****)** based on assuming our estimated prevalence is only for non-hospitalized individuals and assuming the prevalence for hospitalized individuals ranged from 50 to 90% as indicated by existing studies^6–11^.

1 $$\begin{aligned} Prevalence\;of\;Long\;COVID & = \% Unhospitalized \times Prevalence\;among\;the\;Unhospitalized \\ & \quad + \% Hospitalized \times Prevalence\;among\;the\;Hospitalized \\ \end{aligned}$$

Then, we analyzed symptoms connected to long COVID. First, we compared the proportions of our analytical sample who reported each of the symptoms at pre-infection, infection, and post-infection to show the overall recovery from symptoms within the infected population. After that we compared the proportions of those with long COVID in our sample who reported each of the symptoms at each stage to show how their symptoms changed over time, and the frequency of reported symptoms. We also showed the ranking of new-onset persistent symptoms among those with long COVID.

A multivariate logistic regression model was used to identify sociodemographic and health-related risk factors associated with long COVID.

We applied sample weights provided by the UAS to correct for differential response and make our estimates representative of the U.S. population. All analyses were performed using STATA Version 16.0. UAS members have been informed about the survey and consented to be invited to participate in online surveys to provide survey responses. The UAS project was approved by the University of Southern California IRB (UP-14-00148) and complied with the provisions of the Declaration of Helsinki. The current analysis used the STROBE (STrengthening the Reporting of OBservational studies in Epidemiology) cohort reporting guidelines.

## Results

### Sample characteristics at the time of infection

Table 1 shows sample characteristics at the time of reported SARS-CoV-2 infection. Our final sample had a mean age of 46 (third column of Table 1); More than half of the sample was female (57%, 199 respondents); 61% (203) was non-Hispanic White; 12% (25) were non-Hispanic Black; and 22% (56) were Hispanic. Both our final sample and the UAS sample of all COVID cases (the second column of Table 1) are very similar in age, gender, and racial/ethnic distribution to COVID cases tracked by the CDC during the same timeframe^35^ (the first column of Table 1). The unweighted number of respondents corresponding to the proportions can be found in Supplementary Table S2.

**Table 1:** Baseline sample characteristics.

Covariates	CDC COVID cases	COVID population in UAS	Final sample	People with long COVID	People without long COVID	P values
	n = 21,926,390	n = 872	n = 308	n = 74	n = 234	n = 308
	%	%/mean (SD)	%/mean (SD)	%/mean (SD)	%/mean (SD)	With vs without long COVID
Age in years (18–110)	–	45.9 (15.4) IQR = 24	46.0 (15.8) IQR = 26	44.9 (13.9) IQR = 21	46.4 (16.3) IQR = 28	0.555
**Age groups**						
18–49	60.7	60.2	56.5	56.8	56.5	0.973
50–64	23.2	27.4	29.9	34.4	28.6	0.498
65 +	16.1	12.4	13.6	8.9	14.9	0.209
**Gender**						
Male	47.8	44.0	42.7	34.9	45.0	0.270
Female	52.2	56.0	57.3	65.1	55.0	0.270
**Race/ethnicity**						
Non-Hispanic white	55.9	55.7	60.6	62.3	60.1	0.819
Non-Hispanic black	12.2	11.5	12.2	4.1	14.5	0.190
Hispanic	20.7	24.0	22.4	30.0	20.1	0.271
Non-Hispanic others	11.3	8.8	4.9	3.6	5.3	0.586
**Education**						
High school and less	–	39.4	40.9	32.3	43.4	0.253
Some college	–	32.6	35.1	39.0	34.0	0.553
College and more	–	28.0	24.0	28.7	22.6	0.424
**Current smoker**	–	24.8	29.4	19.7	32.2	0.106
**Health conditions**						
Diabetes	–	13.6	17.7	20.0	17.1	0.694
Cancer	–	4.8	5.2	1.1	6.4	0.059
Heart disease	–	6.7	9.2	7.2	9.8	0.540
Hypertension	–	30.7	28.6	33.3	27.2	0.468
Asthma	–	15.3	18.9	24.1	17.3	0.372
Chronic lung disease	–	4.2	4.6	6.8	4.0	0.850
Kidney disease	–	3.5	4.4	7.5	3.5	0.363
Autoimmune disorder	–	5.1	4.7	9.2	3.4	0.055
Obesity	–	20.1	24.2	**42.4**	**18.9**	0.004**
**New-onset symptoms at infection stage**						
Body aches	–	–	44.5	50.3	42.8	0.413
Fatigue	–	–	43.0	47.1	41.7	0.554
Cough	–	–	40.8	42.7	40.3	0.791
Headache	–	–	40.4	**60.0**	**34.7**	0.004**
Fever	–	–	37.3	**51.7**	**33.1**	0.037*
Runny or stuffy nose	–	–	34.8	**49.3**	**30.6**	0.034*
Loss of smell	–	–	32.7	43.5	29.6	0.110
Diarrhea	–	–	28.5	37.7	25.9	0.168
Sore throat	–	–	28.0	40.1	24.5	0.061
Shortness of breath	–	–	26.0	34.9	23.4	0.177
Chest congestion	–	–	25.2	17.2	27.6	0.116
Sneezing	–	–	24.1	33.8	21.2	0.104
> 100.4 °F	–	–	22.7	19.6	23.6	0.616
Abdominal discomfort	–	–	22.3	30.1	20.0	0.187
Dry skin	–	–	11.2	15.6	10.0	0.327
Vomiting	–	–	7.8	13.7	6.1	0.167
Skin rash	–	–	4.0	3.7	4.1	0.903
Hair loss	–	–	2.6	6.3	1.5	0.100
Symptomatic at infection	–	83.2	80.3	100.0	74.6	–
Symptom count at infection (0–18)	–	5.8 (4.6) IQR = 8	6.0 (4.6) IQR = 9	7.9 (3.3) IQR = 5	5.4 (4.7) IQR = 9	0.000***

The CDC COVID cases information were from CDC COVID Data Tracker: https://covid.cdc.gov/covid-data-tracker/#demographics.

For the UAS sample, the time of COVID diagnosis is considered baseline, and all percentages and means are weighted to be nationally representative.

Significant values are in bold.

*p < 0.05, **p < 0.01, ***p < 0.001.

In the final sample, about 41% (73) had high school or less than high school completion, 35% (139) reported some college, and 24% (96) had a bachelor’s degree or higher. Almost 30% (74) of the respondents were current smokers. In terms of existing health conditions, 18% (50) had diabetes, 5% (17) had cancer, 9% (26) had heart disease, 29% (97) had high blood pressure, 19% (47) had asthma, 5% (15) had chronic lung disease, 4% (13) had kidney disease, 5% (25) had an autoimmune disorder, and 24% (78) were obese. Half of the sample had none of the underlying conditions.

More than two fifths of the sample reported new-onset body aches (45%, 128), fatigue (43%, 124), cough (41%, 119), and headache (40%, 116) at the infection stage; in addition, more than one fourth had new-onset fever (37%, 109), runny or stuffy nose (35%, 119), loss of smell (33%, 100), diarrhea (29%, 72), sore throat (28%, 77), shortness of breath (26%, 62), and chest congestion (25%, 75).

At the time of infection, 80% (254) of the respondents were symptomatic, and the average symptom count was 6. Both the proportion symptomatic and the average symptom count were fairly similar between the UAS total COVID sample (the second column of Table 1) and our final analytical sample (the third column of Table 1). Persons with long COVID had more symptoms on average than those who recovered quickly (7.9 vs 5.4).

Compared to people who did not experience long COVID, the long haulers were significantly more likely to be obese (p = 0.004). In terms of new-onset symptoms, the long haulers were more likely to experience headache (0.004), fever (0.037), and runny or stuffy nose (0.034).

### The prevalence of long COVID

In our sample of 308 COVID-infected respondents, 40% (132 respondents) experienced at least one symptom 12 weeks after reporting COVID, and this would have been the estimated prevalence of long COVID if pre-infection symptoms were not considered. However, after accounting for pre-infection symptoms, only 23% (74 respondents) of the infected experienced at least one new-onset COVID symptom that lasted for at least 12 weeks. These estimates can be compared to the other nationally representative prevalence of long COVID estimated for UK by ONS and England by Whitaker et al. of 10% and 38% respectively. Our long COVID prevalence (40%) among the U.S. population without accounting for pre-infection symptom level, is very similar to that made by Whitaker et al. of 38%. and after controlling for pre-infection symptoms, our estimate is between the other two.

We are likely to have missed some severe COVID cases who were unlikely to have answered the survey while suffering from severe illness. Given that around 5% of the SARS-CoV-2 infected population are hospitalized^12^, and since long COVID is highly prevalent (50%-90%) among hospitalized patients^6–11^, we believe that the real prevalence for the U.S. adult population is likely higher than our estimate. To determine the prevalence of long COVID accounting for the hospitalized population we use Eq. (1) and assume a prevalence of 23% (our estimate accounting for pre-infection symptoms) for long COVID among non-hospitalized infected individuals and a prevalence ranging from 50 to 90% for those hospitalized with COVID. We first estimate the lower end of the prevalence of long COVID in the population under the assumption that 50% of those hospitalized will have long COVID: ${(95\%}_{non-hopitalized}\times 0.23)+\left({5\%}_{hospitalized}\times 0.5\right)=0.243 or 24\%$. We then estimate the upper end of the prevalence of long COVID in the population under the assumption that 90% of those hospitalized will have long COVID: ${(95\%}_{non-hopitalized}\times 0.23)+\left({5\%}_{hospitalized}\times 0.9\right)=0.263 or 26\%$. Thus, we believe that the real prevalence for the U.S. adult population would range from 24 to 26%, which is slightly higher than our estimate of 23%.

### Symptoms trend and most reported symptoms

**Figure 1:**
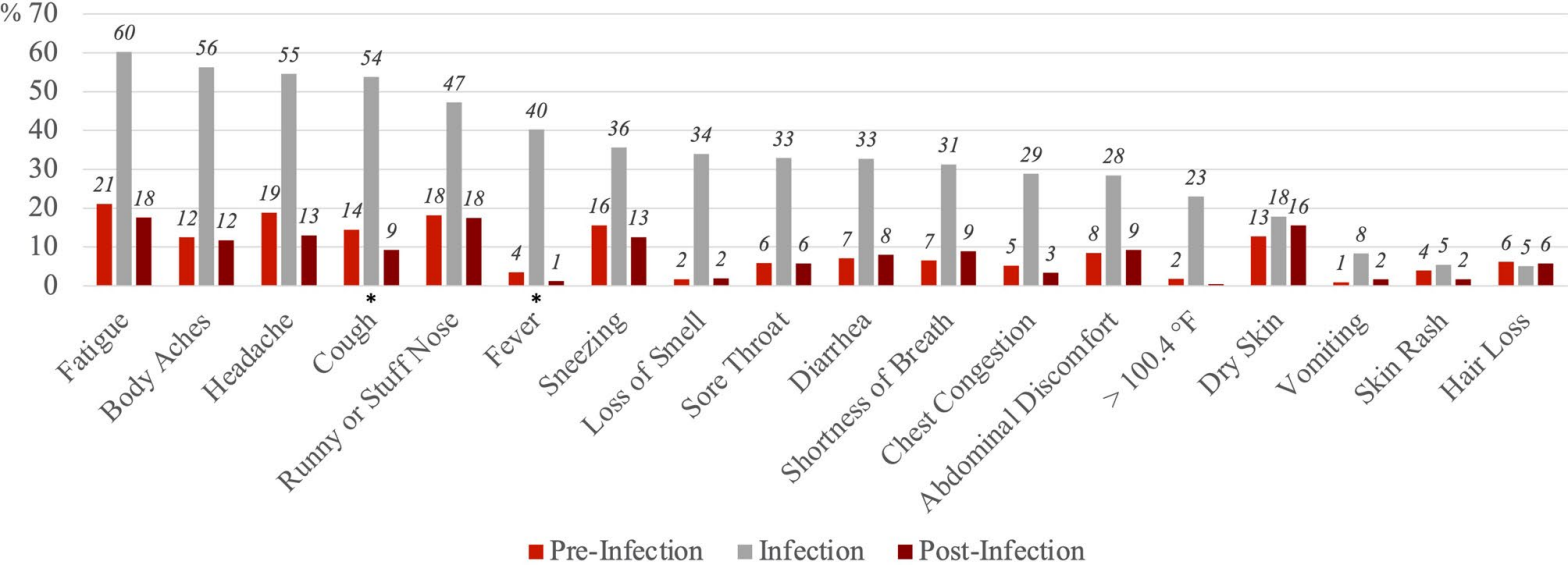
Percent with self-reported symptoms at pre-infection, infection, and post-infection stages, among the infected (n = 308). The pre-infection stage is 4 weeks before the COVID diagnosis or positive test. The infection stage is the time of COVID diagnosis or positive test. The post-infection stage is 12 weeks after the COVID diagnosis or positive test. Symptoms were listed based on the proportion reported at the time of infection. Wald (χ^2^) tests were used to determine statistically significant differences in symptoms at the pre-infection stage and post-infection stage, and standard errors were clustered at the individual level. *p < 0.05, **p < 0.01, ***p < 0.001.

Figure 1 shows the proportions of our sample reporting each of the symptoms at pre-infection, infection, and post-infection stages. Among the infected, more than half experienced fatigue (60%), body aches (56%), headache (55%), and cough (54%) at the time of infection. For most of the symptoms, the proportions are elevated at the time of infection, but overall, tended to return to pre-infection levels at the post-infection stage.

**Figure 2:**
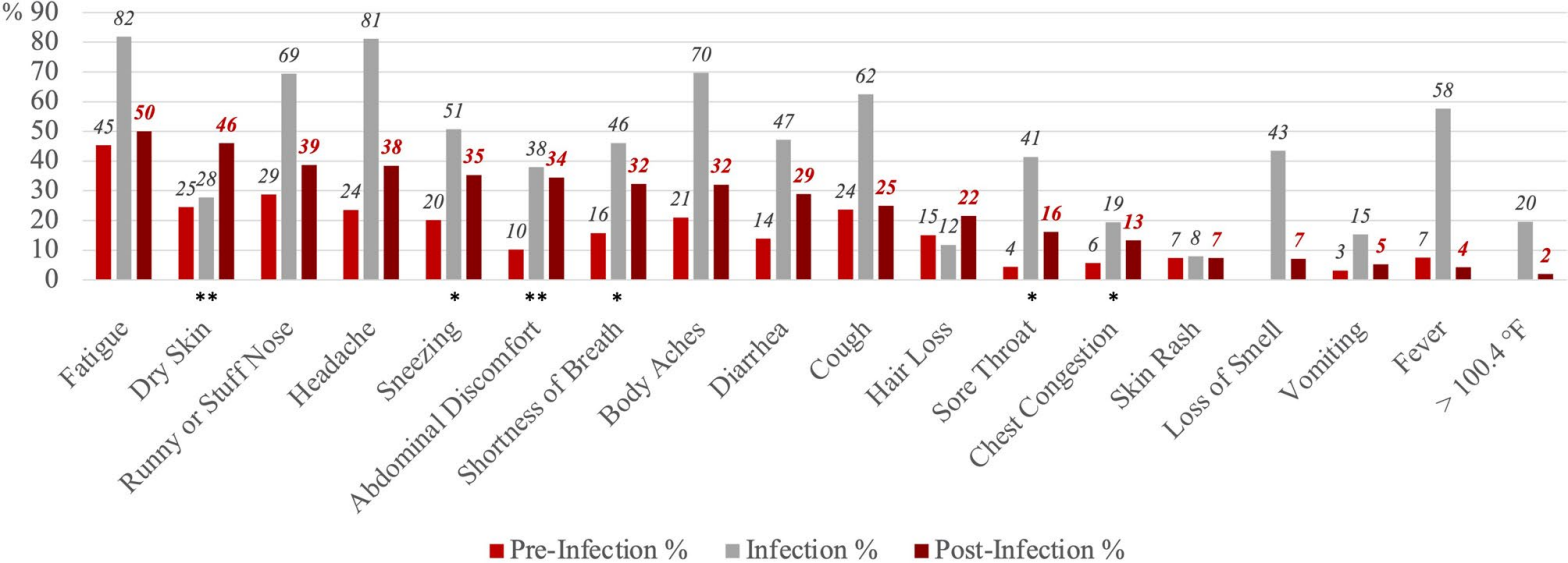
Percent with self-reported symptoms at pre-infection, infection, and post-infection stages among COVID long haulers (n = 74). The pre-infection stage is 4 weeks before the COVID diagnosis or positive test. The infection stage is the time of COVID diagnosis or positive test. The post-infection stage is 12 weeks after the COVID diagnosis or positive test. Symptoms were listed based on the proportion reported at the post-infection stage. Wald (χ^2^) tests were used to determine statistically significant differences in symptoms at the pre-infection stage and post-infection stage, and standard errors were clustered at the individual level. *p < 0.05, **p < 0.01, ***p < 0.001.

Similarly, Fig. 2 shows the proportions reporting each of the symptoms at the three stages, but only among the COVID long haulers (n = 74). For many symptoms, the proportions peaked at the time of infection and then dropped but remained higher at the post-infection stage compared to pre-infection stage. Specifically, relative to the pre-infection level, the proportion reporting dry skin (p = 0.006), sneezing (p = 0.020), abdominal discomfort (p = 0.003), shortness of breath (p = 0.020), sore throat (p = 0.039), and chest congestion (p = 0.047) were statistically significantly higher at the post-infection stage based on Wald (χ^2^) tests (standard errors are clustered at the individual level). Among those with long COVID, the most commonly reported symptoms at the post-infection stage included fatigue (50%), dry skin (46%), runny or stuffy nose (39%), headache (38%), and sneezing (35%). It is important to note that these most reported symptoms did not account for pre-infection baseline level, and it is possible that they were commonly reported partially because they had high prevalence even without SARS-CoV-2 infection. The percent with self-reported symptoms at the three stages among those infected non-long-haulers can be found in Supplementary Fig. S1.

**Figure 3:**
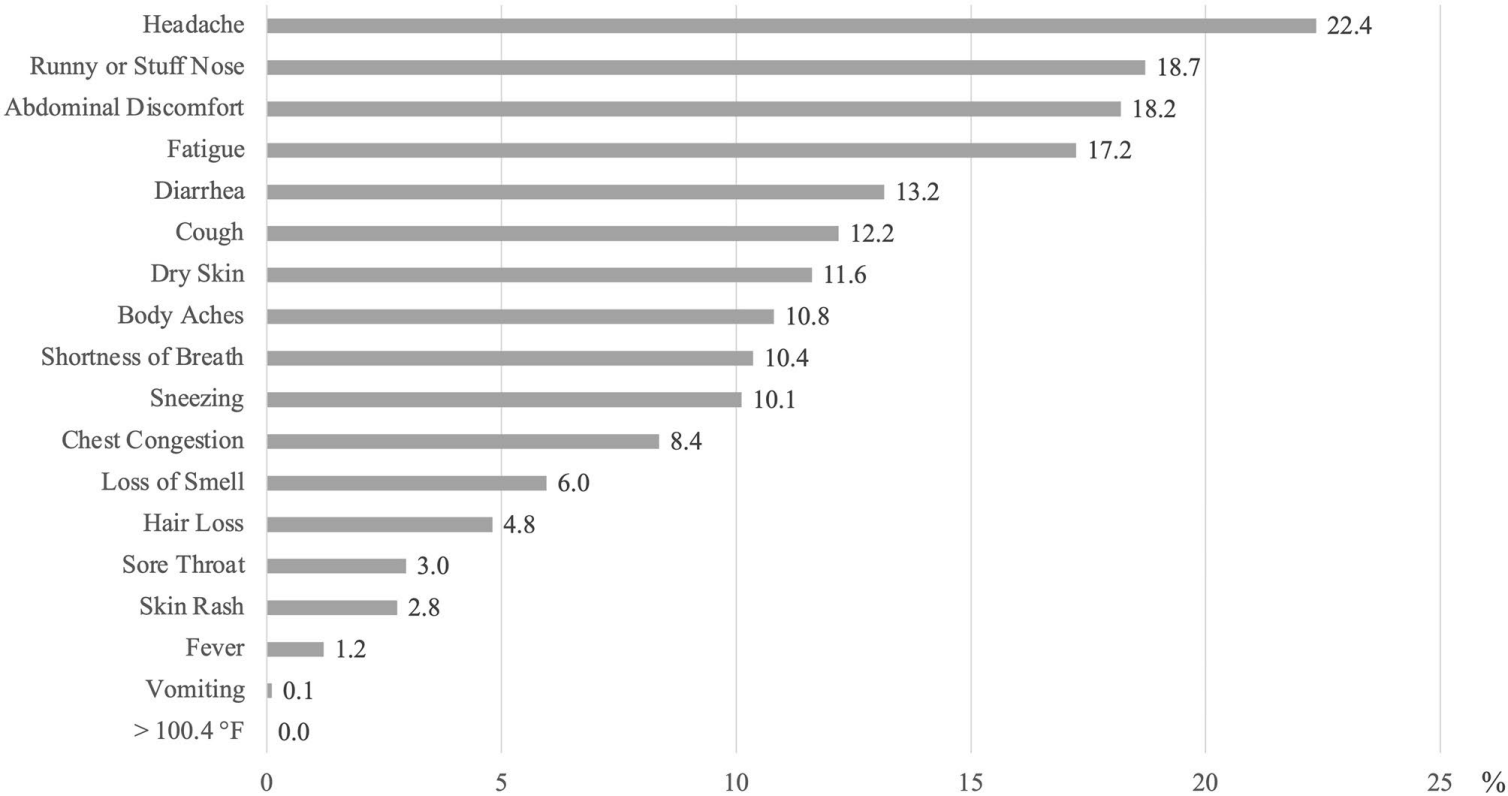
Prevalence of new-onset persistent COVID symptoms among those with long COVID 12 weeks after infection.

To account for the pre-infection prevalence of the symptoms, Fig. 3 shows the prevalence of only new-onset persistent symptoms among those with long COVID at the post-infection stage. Because the long haulers started experiencing these symptoms at the time of infection, they are more likely to be related to COVID specifically. The most reported new-onset persistent symptoms were headache (22%), runny or stuffy nose (19%), abdominal discomfort (18%), fatigue (17%), and diarrhea (13%). In terms of both the ranking and the prevalence, many symptoms in Fig. 3 are different from Fig. 2. For example, the rankings of dry skin (No.2 to No.7) and sneezing (No.5 to No.10) dropped markedly, while the rankings of diarrhea (No.9 to No.5) and cough (No.10 to No.6) notably increased.

### Predictors of long COVID

Table 2 shows the logistic regression model predicting long COVID among our sample of 308 SARS-CoV-2 infected respondents. Since fever at the infection stage is one of the potential predictors, we exclude body temperature higher than 100.4 °F during the infection stage from our model to avoid multicollinearity (those two variables have a correlation coefficient of 0.65). People who were obese (OR = 5.44, 95% CI 2.12–13.96), and who experienced hair loss (OR = 6.94, 95% CI 1.03–46.92), headache (OR = 3.37, 95% CI 1.18–9.60), and sore throat (OR = 3.56, 95% CI 1.21–10.46) at the time of infection, had significantly higher odds of experiencing long COVID. On the contrary, the odds among people who experienced chest congestion (OR = 0.09, 95% CI 0.02–0.35) were lower. None of the existing chronic health conditions were related to having long COVID. The odds were not significantly different across demographic and education groups in either the full model or the model unadjusted for other covariates.

**Table 2:** Logistic regression model predicting long COVID.

n = 308	Odds Ratio	95% CI
**Age categories—Ref: 18–44**		
45–64	0.76	[0.27, 2.13]
65 +	0.94	[0.26, 3.38]
Male	0.93	[0.46, 1.91]
**Race/ethnicity—Ref: non-hispanic white**		
Non-Hispanic black	0.46	[0.07, 3.07]
Hispanic	0.72	[0.30, 1.72]
Non-Hispanic others	0.50	[0.08, 3.25]
**Education categories—Ref: high school and less**		
Some college	1.45	[0.60, 3.55]
College and more	1.38	[0.50, 3.79]
**Current Smoker**	0.74	[0.28, 1.94]
**Existing conditions**		
Obesity	5.44***	[2.12, 13.96]
Diabetes	1.03	[0.30, 3.48]
Cancer	0.10	[0.01, 1.16]
Heart disease	0.21	[0.03, 1.48]
High blood pressure	1.38	[0.55, 3.43]
Asthma	0.96	[0.26, 3.62]
Chronic lung disease	3.05	[0.18, 52.77]
Kidney disease	1.28	[0.19, 8.68]
Autoimmune disorder	1.83	[0.51, 6.62]
**New-onset symptoms at infection stage**		
Body aches	1.25	[0.42, 3.74]
Fatigue	0.46	[0.15, 1.45]
Cough	0.54	[0.19, 1.52]
Headache	3.37*	[1.18, 9.60]
Fever	1.06	[0.40, 2.83]
Runny or stuffy nose	1.38	[0.49, 3.84]
Loss of smell	1.58	[0.72, 3.50]
Diarrhea	1.02	[0.46, 2.27]
Sore throat	3.56*	[1.21, 10.46]
Shortness of breath	1.70	[0.52, 5.58]
Chest congestion	0.09***	[0.02, 0.35]
Sneezing	1.56	[0.58, 4.24]
Abdominal discomfort	1.26	[0.54, 2.93]
Dryskin	1.10	[0.29, 4.18]
Vomiting	0.75	[0.22, 2.64]
Skin rash	0.53	[0.11, 2.55]
Hairloss	6.94*	[1.03, 46.92]

Coefficients are reported in odds ratios.

95% confidence intervals are reported in brackets.

*p < 0.05, **p < 0.01, ***p < 0.001.

## Discussion

### Main findings

Our results indicate that the estimated prevalence of long COVID in a population representative sample differs depending on whether pre-infection symptoms are accounted for. In the U.S. population, most people with COVID return to their pre-infection symptom level after the acute phase of the disease. However, more than one-fifth (23%) experience long COVID, with at least one symptom originating around the time of SARS-CoV-2 infection lasting for more than 12 weeks. Without adjusting for pre-infection symptoms, the prevalence is estimated to be 40%, which suggests the potential for a significant over-estimation of long COVID in previous studies.

The most frequently experienced new-onset persistent symptoms among those with long COVID include headache (22%), runny or stuffy nose (19%), abdominal discomfort (18%), fatigue (17%), and diarrhea (13%). The fully adjusted logistic regression model indicates that the likelihood of experiencing long COVID is not significantly associated with sociodemographic or behavioral factors including age, gender, race/ethnicity, education, current smoking status or the presence of chronic conditions. COVID long haulers are more likely to experience hair loss, headache, and sore throat at the time of infection compared to their counterparts whose symptoms reduce more quickly. Also, those who are obese are at higher risk of experiencing new-onset persistent symptoms.

To our knowledge, this is the first study that defined long COVID accounting for pre-infection baseline symptoms using longitudinal data. More than two-fifths (44%) of our sample reported experiencing at least one symptom prior to infection, which were likely due to other non-COVID conditions. So, while around 40% of the COVID-infected had at least one symptom 12 weeks after COVID infection, this may overestimate the prevalence of long COVID if these symptoms were occurring prior to COVID infection. Our study used longitudinal data on individuals observed from pre-infection to post-infection stage, which made it possible to distinguish new onset symptoms from the symptoms that might be experienced by someone without SARS-CoV-2 infection.

Compared to estimates of long COVID prevalence based on other nationally representative studies, our estimate (23%) based on UAS data is between the U.K. ONS estimate (10%)^23^, and the Whitaker et al. estimate (38%)^24^. The three studies are similar in study design and population representativeness, so the difference in estimates may reflect the different number of symptoms used in each study. Specifically, the ONS estimate is based on 12 symptoms, while the Whitaker et al. estimate on 29 symptoms. The current study included 18 symptoms, which is roughly between the other two studies. The symptoms included in Whitaker et al.’s study but not in UAS include sudden swelling to face or lips, sore eyes, purple scores/blisters on feet, numbness/tingling, hoarse voice, heavy arms/legs, dizziness, difficulty sleeping, chills, and appetite loss. However, these symptoms generally have low prevalence among the SARS-CoV-2 infected, and/or diminish quickly after initial infection^24^. Hence, the lack of these symptoms in the questionnaire is not likely to be a source of a significant difference in the estimated prevalence of long COVID.

Our estimated prevalence is also similar to the estimate of 27% based on never-hospitalized COVID symptomatic Californians^17^, the estimate of 30% based on a sample combining hospitalized patients and outpatients in Seattle, Washington^1^, and the meta-analysis estimate of 34% based on studies focusing on only non-hospitalized individuals^21^. While it is notably lower than the estimated prevalence of at least 50% using a hospitalized patient sample in Michigan^8^. These differences may reflect the fact that we adjust for pre-infection symptoms, and we probably underrepresent the hospitalized population.

The significant association between long COVID and obesity is consistent with previous studies^24,27,28^. Both Whitaker et al.’s and the ONS studies found that existing health conditions are associated with elevated long COVID risk; while our results do not show any link between the presence of health conditions and long COVID.

We differ from some existing studies, in that we did not find a significant association between long COVID and any sociodemographic factors included in this study. It is probably because the analytic approaches used by the ONS^23^, Whitaker et al.^24^, and Sudre et al.^27^ to assess risk factors for long COVID either are based on bivariate comparisons, or do not include the effects of existing health conditions as we do. Hence, the age differences and gender differences they found may be explained by health differences across gender and age groups or other uncontrolled factors. Also, Sudre et al. collected data from an international sample including respondents from the U.K., the U.S., and Sweden, while Whitaker et al. focused on England and the ONS focused on the U.K. population. The discrepancy in results may also reflect differences in socioeconomic and demographic context across countries. We found some symptoms reported at the time of infection to be associated with experiencing long COVID, but the symptoms we found (hair loss, headache, and sore throat) are different from the ones identified by Augustin et al.^16^ (anosmia and diarrhea). It is probably because we used new-onset symptoms as the predictors in our regression model, but the previous study was not able to distinguish new-onset symptoms from those started even before SARS-CoV-2 infection.

We did not include vaccination status in the current analysis because vaccines were not widely available during our study period. Only 17 individuals had received at least one dose of the COVID vaccine during the follow-up period in our study. We examined symptoms trajectories in these cases and did not find any notable differences in their symptoms post vaccine and the prevalence of long COVID among these 17 was not statistically significantly different from others.

### Limitations

Our study has several limitations. Though we utilized the longitudinal nature of the data to account for pre-infection symptoms, admittedly, this approach only identifies new-onset symptoms and not the changing severity of symptoms. It also does not consider long COVID cases where the symptoms are absent at the infection stage but arise later which would result in an underestimated prevalence of long COVID. However, since the primary goal is to reveal the difference in estimated prevalence with and without adjusting for pre-infection symptoms, our longitudinal and conservative approach does this while avoiding possible overestimation.

Some limitations of our study are due to the nature of the secondary data we use. The UAS COVID National Survey has a large panel, but limiting our analysis to those with SARS-CoV-2 infection and requiring data on symptoms both before and after infection further results in a small sample size. This may limit the statistical power of our model, and thus the associations, especially those based on smaller population subgroups such as non-Hispanic others and those with less common existing conditions and symptoms, should be interpreted with caution. In addition, the UAS study does not have information on some other symptoms that are potentially related to long COVID, such as brain fog, cognitive impairment, and hoarse voice. Thus, we may have underestimated COVID long haulers who suffered from only these symptoms.

Finally, the latest possible date of positive test or diagnosis in our study was between November 25 and December 23, 2020. This dating means we did not need to consider disease variants in interpreting our findings. Specifically, since most COVID variants did not start circulating in the US until 2021^36^, the presence of new variants was not a factor in our results. However, with the availability of vaccines and the onset of new variants, the nation has moved into new stages of the pandemic. The vaccinated population has tripled since the end of our study period, and by March 2022, more than 65% of the total US population have been fully vaccinated^37^. The later variants beginning with the Omicron variant spread more easily than the original virus. Also, it remains unclear how vaccination affects long COVID under the new context^4^, and there is limited evidence on whether the Omicron wave has changed what we know about long COVID^38,39^. Nevertheless, long COVID is a major public health concern. More knowledge on its prevalence, persistent symptoms, and risk factors may help healthcare professionals allocate resources and services to help long haulers get back to normal lives.

## Supplementary Information

Supplementary Information.

## Data Availability

The survey and data are available from the University of Southern California Understanding America Study website: https://uasdata.usc.edu/index.php.

## References

[1] Logue JK, Franko NM, McCulloch DJ, McDonald D, Magedson A, Wolf CR (2021). Sequelae in adults at 6 months after COVID-19 infection. JAMA Netw. Open..

[2] Venkatesan, P. NICE guideline on long COVID. *Lancet Respir. Med*. **9**(2), 129. 10.1016/S2213-2600(21)00031-X (2021). 10.1016/S2213-2600(21)00031-X PMC7832375 33453162

[3] Collins F. NIH launches new initiative to study “Long COVID”. https://www.nih.gov/about-nih/who-we-are/nih-director/statements/nih-launches-new-initiative-study-long-covid (2021).

[4] Centers for Disease Control and Prevention. Post-COVID Conditions. https://www.cdc.gov/coronavirus/2019-ncov/long-term-effects/index.html (2021).

[5] World Health Organization. A clinical case definition of post COVID-19 condition by a delphi consensus. https://www.who.int/publications/i/item/WHO-2019-nCoV-Post_COVID-19_condition-Clinical_case_definition-2021.1 (2021).

[6] Carfì A, Bernabei R, Landi F (2020). Persistent symptoms in patients after acute COVID-19. JAMA.

[7] Garrigues E, Janvier P, Kherabi Y, Le Bot A, Hamon A, Gouze H (2020). Post-discharge persistent symptoms and health-related quality of life after hospitalization for COVID-19. J Infect..

[8] Chopra V, Flanders SA, O’Malley M, Malani AN, Prescott HC (2021). Sixty-day outcomes among patients hospitalized with COVID-19. Ann. Int. Med..

[9] Halpin SJ, McIvor C, Whyatt G, Adams A, Harvey O, McLean L (2021). Postdischarge symptoms and rehabilitation needs in survivors of COVID-19 infection: A cross-sectional evaluation. J. Med. Virol..

[10] Huang C, Huang L, Wang Y, Li X, Ren L, Gu X (2021). 6-month consequences of COVID-19 in patients discharged from hospital: a cohort study. The Lancet..

[11] Zhao Y, Shang Y, Song W, Li Q, Xie H, Xu Q (2020). Follow-up study of the pulmonary function and related physiological characteristics of COVID-19 survivors three months after recovery. Clin. Med..

[12] Centers for Disease Control and Prevention. Estimated COVID-19 Burden. https://www.cdc.gov/coronavirus/2019-ncov/cases-updates/burden.html (2021).

[13] Carvalho-Schneider C, Laurent E, Lemaignen A, Beaufils E, Bourbao-Tournois C, Laribi S (2021). Follow-up of adults with noncritical COVID-19 two months after symptom onset. Clin. Microbiol. Infect..

[14] Seeßle J, Waterboer T, Hippchen T, Simon J, Kirchner M, Lim A (2021). Persistent symptoms in adult patients one year after COVID-19: a prospective cohort study. Clin. Infect. Dis..

[15] Blomberg B, Mohn KGI, Brokstad KA, Zhou F, Linchausen DW, Hansen B-A (2021). Long COVID in a prospective cohort of home-isolated patients. Nat. Med..

[16] Augustin M, Schommers P, Stecher M, Dewald F, Gieselmann L, Gruell H (2021). Post-COVID syndrome in non-hospitalised patients with COVID-19: A longitudinal prospective cohort study. Lancet Region. Health-Eur..

[17] Huang, Y., Pinto, M.D., Borelli, J.L., Mehrabadi, M.A., Abrihim, H., & Dutt, N., et al. COVID Symptoms, Symptom Clusters, and Predictors for Becoming a Long-Hauler: Looking for Clarity in the Haze of the Pandemic. *medRxiv*. 2021.03.03.21252086. 10.1101/2021.03.03.21252086 (2021). 10.1177/10547738221125632 PMC9510954 36154716

[18] Tenforde MW, Kim SS, Lindsell CJ, Billig Rose E, Shapiro NI, Files DC (2020). Symptom duration and risk factors for delayed return to usual health among outpatients with COVID-19 in a multistate health care systems network—United States, March–June 2020. MMWR Morb. Mortal Wkly Rep..

[19] Klein H, Asseo K, Karni N, Benjamini Y, Nir-Paz R, Muszkat M (2021). Onset, duration and unresolved symptoms, including smell and taste changes, in mild COVID-19 infection: A cohort study in Israeli patients. Clin. Microbiol. Infect..

[20] Taquet M, Dercon Q, Luciano S, Geddes JR, Husain M, Harrison PJ (2021). Incidence, co-occurrence, and evolution of long-COVID features: A 6-month retrospective cohort study of 273,618 survivors of COVID-19. PLOS Med..

[21] Chen C, Haupert SR, Zimmermann L, Shi X, Fritsche LG, Mukherjee B (2022). Global prevalence of post COVID-19 condition or long COVID: A meta-analysis and systematic review. J. Infect. Dis..

[22] Fernández-de-Las-Peñas C, Palacios-Ceña D, Gómez-Mayordomo V, Florencio LL, Cuadrado ML, Plaza-Manzano, (2021). Prevalence of post-COVID-19 symptoms in hospitalized and non-hospitalized COVID-19 survivors: A systematic review and meta-analysis. Eur. J. Intern. Med..

[23] Office for National Statistics. The prevalence of long COVID symptoms and COVID-19 complications. https://www.ons.gov.uk/news/statementsandletters/theprevalenceoflongcovidsymptomsandcovid19complications (2020).

[24] Whitaker, M., Elliott, J., Chadeau-Hyam, M., Riley, S., Darzi, A., & Cooke, G, et al. Persistent symptoms following SARS-CoV-2 infection in a random community sample of 508,707 people. *medRxiv*. 2021.06.28.21259452. 10.1101/2021.06.28.21259452 (2021).

[25] Centers for Disease Control and Prevention. Post-COVID Conditions: Information for Healthcare Providers. https://www.cdc.gov/coronavirus/2019-ncov/hcp/clinical-care/post-covid-conditions.html?CDC_AA_refVal=https%3A%2F%2Fwww.cdc.gov%2Fcoronavirus%2F2019-ncov%2Fhcp%2Fclinical-care%2Flate-sequelae.html (2021).

[26] National Health Service. Long-term effects of coronavirus (long COVID). https://www.nhs.uk/conditions/coronavirus-covid-19/long-term-effects-of-coronavirus-long-covid/ (2021).

[27] Sudre, C.H., Murray, B., Varsavsky, T., Graham, M.S., Penfold, R.S., & Bowyer, R.C., et al. Attributes and predictors of long COVID. *Nat. Med*. 1–6. 10.1038/s41591-021-01292-y (2021). 10.1038/s41591-021-01292-y PMC7611399 33692530

[28] Office for National Statistics. Prevalence of ongoing symptoms following coronavirus (COVID-19) infection in the UK: 4 June 2021. https://www.ons.gov.uk/peoplepopulationandcommunity/healthandsocialcare/conditionsanddiseases/bulletins/prevalenceofongoingsymptomsfollowingcoronaviruscovid19infectionintheuk/4june2021 (2021).

[29] Thompson, E.J., Williams, D.M., Walker, A.J., Mitchell, R.E., Niedzwiedz, C.L., & Yang, T.C., et al. Risk factors for long COVID: analyses of 10 longitudinal studies and electronic health records in the UK. *medRxiv*. 2021.06.24.21259277. 10.1101/2021.06.24.21259277 (2021).

[30] Kompaniyets, L., Pennington, A.F., Goodman, A.B., Rosenblum, H.G., Belay, B., & Ko, J.Y., et al. Underlying medical conditions and severe illness among 540,667 adults hospitalized with COVID-19, March 2020–March 2021. *Prev. Chronic Dis*. **18**, E66. 10.5888/pcd18.210123 (2021). 10.5888/pcd18.210123 PMC8269743 34197283

[31] Centers for Disease Control and Prevention. Risk of Exposure to COVID-19. https://www.cdc.gov/coronavirus/2019-ncov/community/health-equity/racial-ethnic-disparities/increased-risk-exposure.html (2020).

[32] Center for Economic and Social Research. About the UAS. https://uasdata.usc.edu/index.php (2017).

[33] Center for Economic and Social Research. Understanding Coronavirus in America – Survey Methods. https://covid19pulse.usc.edu/?methods (2020).

[34] Centers for Disease Control and Prevention. People with Certain Medical Conditions. https://www.cdc.gov/coronavirus/2019-ncov/need-extra-precautions/people-with-medical-conditions.html (2021).

[35] Centers for Disease Control and Prevention. Demographic Trends of COVID-19 cases and deaths in the US reported to CDC. https://covid.cdc.gov/covid-data-tracker/#demographics (2021).

[36] Galloway SE, Paul P, MacCannell DR, Johansson MA, Brooks JT, MacNeil A, et. al. Emergence of SARS-CoV-2 b. 1.1. 7 lineage—united states, December 29, 2020–January 12, 2021. *Morbid. Mortal. Week. Rep.* 70(3), 95. 10.15585/mmwr.mm7003e2 (2021) 10.15585/mmwr.mm7003e2 PMC7821772 33476315

[37] Centers for Disease Control and Prevention. COVID-19 Vaccinations in the United States. https://covid.cdc.gov/covid-data-tracker/#vaccinations_vacc-total-admin-rate-total (2022).

[38] The Harvard Gazette. Hints of a long COVID wave as Omicron fades. https://news.harvard.edu/gazette/story/2022/02/harvard-experts-expect-new-wave-of-long-covid-cases/ (2022).

[39] Tayag, Y. What causes long Covid? Scientists are zeroing in on the answer. https://www.vox.com/22906853/omicron-long-covid-vaccinated-symptoms-cause (2022).

