# Supplementary material for: Long COVID and symptom trajectory in a representative sample of Americans in the first year of the pandemic
Source: Sci Rep. 2022 Jul 8;12:11647. doi: 10.1038/s41598-022-15727-0 (PMC9264749; doi:10.1038/s41598-022-15727-0)
Supplement: Supplementary file 1 — Supplementary Information. [file 41598_2022_15727_MOESM1_ESM.pdf]

Long COVID and Symptom Trajectory in a Representative Sample of Americans in the First Year of the Pandemic

Qiao Wu*[1], Jennifer A. Ailshire[2], and Eileen M. Crimmins[2]

[1] MIPM, Leonard Davis School of Gerontology, University of Southern California, Los Angeles, California, The United States

[2] PhD, Leonard Davis School of Gerontology, University of Southern California, Los Angeles, California, The United States.

*Corresponding Author: Qiao Wu

University of Southern California, Andrus Gerontology Center, 3715 McClintock Ave, Room 215, Los Angeles, California 90089-0191

+1 2137067258 | qiaowu@usc.edu

Supplementary Information

Supplementary Table 1. Difference between Final Sample and Dropped Participants

Covariates	**Final Sample** n = 308 % / *mean (SD)*	**Dropped** n = 564 % / *mean (SD)*	**T-Test P Values** n = 308 Final Sample VS Dropped
**Age in Years (18-110)**	*46.0 (15.8)* *IQR=26*	*45.8 (15.2)* *IQR=23*	0.897
**Age Groups**			
18-49	56.5	62.1	0.229
50-64	29.9	26.1	0.366
65+	13.6	11.8	0.537
**Gender**			
Male	42.7	44.7	0.674
Female	57.3	55.3	0.674
**Race/Ethnicity**			
Non-Hispanic White	60.6	53.1	0.136
Non-Hispanic Black	12.2	11.1	0.757
Hispanic	22.4	24.9	0.588
Non-Hispanic Others	4.9	10.9	**0.031***
**Education**			
High School and Less	40.9	38.7	0.647
Some College	35.1	31.3	0.378
College and More	24.0	30.1	0.138
**Current Smoker**	29.4	22.4	0.105
**Health Conditions**			
Diabetes	17.7	11.4	0.059
Cancer	5.2	4.5	0.745
Heart Disease	9.2	5.4	0.116
Hypertension	28.6	31.9	0.451
Asthma	18.9	13.3	0.124
Chronic Lung Disease	4.6	4.0	0.757
Kidney Disease	4.4	3.0	0.468
Autoimmune Disorder	4.7	5.4	0.701
Obesity	24.2	17.8	0.094
**Symptomatic at Infection**	80.3	84.7	0.257
**Symptom Count at Infection (0-18)**	*6.0 (4.6)* *IQR=9*	*5.8 (4.6)* *IQR=8*	0.698

Notes
* p<0.05 ** p<0.01 *** p<0.001

Supplementary Table 2. Baseline Sample Characteristics

Covariates	COVID Population in UAS<br>n = 872<br>% (n) / *mean (SD)*	Final Sample<br>n = 308<br>% (n) / *mean (SD)*	People with Long COVID<br>n = 74<br>% (n) / *mean (SD)*	People without Long COVID<br>n = 234<br>% (n) / *mean (SD)*
**Age in Years (18-110)**	*45.9 (15.4)*<br>*IQR=24*	*46.0 (15.8)*<br>*IQR=26*	*44.9 (13.9)*<br>*IQR=21*	*46.4 (16.3)*<br>*IQR=28*
**Age Groups**				
18-49	60.2 (493)	56.5 (161)	56.8 (39)	56.5 (122)
50-64	27.4 (260)	29.9 (95)	34.4 (24)	28.6 (71)
65+	12.4 (119)	13.6 (52)	8.9 (11)	14.9 (41)
**Gender**				
Male	44 (308)	42.7 (109)	34.9 (22)	45 (87)
Female	56 (564)	57.3 (199)	65.1 (52)	55 (147)
**Race/Ethnicity**				
Non-Hispanic White	55.7 (512)	60.6 (203)	62.3 (51)	60.1 (152)
Non-Hispanic Black	11.5 (66)	12.2 (25)	4.1 (1)	14.5 (24)
Hispanic	24 (207)	22.4 (56)	30 (16)	20.1 (40)
Non-Hispanic Others	8.8 (87)	4.9 (24)	3.6 (6)	5.3 (18)
**Education**				
High School and Less	39.4 (221)	40.9 (73)	32.3 (15)	43.4 (58)
Some College	32.6 (367)	35.1 (139)	39 (39)	34 (100)
College and More	28 (284)	24 (96)	28.7 (20)	22.6 (76)
**Current Smoker**	24.8 (200)	29.4 (74)	19.7 (15)	32.2 (59)
**Health Conditions**				
Diabetes	13.6 (121)	17.7 (50)	20 (15)	17.1 (35)
Cancer	4.8 (50)	5.2 (17)	1.1 (2)	6.4 (15)
Heart Disease	6.7 (55)	9.2 (26)	7.2 (7)	9.8 (19)
Hypertension	30.7 (272)	28.6 (97)	33.3 (25)	27.2 (72)
Asthma	15.2 (122)	18.9 (47)	24.1 (16)	17.3 (31)
Chronic Lung Disease	4.2 (36)	4.6 (15)	6.8 (6)	4 (9)
Kidney Disease	3.5 (26)	4.4 (13)	7.5 (3)	3.5 (10)
Autoimmune Disorder	5.1 (66)	4.7 (25)	9.2 (12)	3.4 (13)
Obesity	20.1 (205)	24.2 (78)	42.4 (29)	18.9 (49)
**New-Onset Symptoms at Infection Stage**				
Body aches	-	44.5 (128)	50.3 (38)	42.8 (90)
Fatigue	-	43 (124)	47.1 (31)	41.7 (93)
Cough	-	40.8 (119)	42.7 (32)	40.3 (87)
Headache	-	40.4 (116)	60 (34)	34.7 (82)
Fever	-	37.3 (109)	51.7 (32)	33.1 (77)
Runny or stuffy nose	-	34.8 (119)	49.3 (35)	30.6 (84)
Lost of smell	-	32.7 (100)	43.5 (30)	29.6 (70)
Diarrhea	-	28.5 (72)	37.7 (27)	25.9 (45)
Sore throat	-	28 (77)	40.1 (25)	24.5 (52)
Shortness of breath	-	26 (62)	34.9 (19)	23.4 (43)
Chest congestion	-	25.2 (75)	17.2 (21)	27.6 (54)
Sneezing	-	24.1 (79)	33.8 (28)	21.2 (51)
> 100.4 °F	-	22.7 (68)	19.6 (15)	23.6 (53)
Abdominal discomfort	-	22.3 (66)	30.1 (25)	20 (41)
Dryskin	-	11.2 (31)	15.6 (12)	10 (19)
Vomiting	-	7.8 (21)	13.7 (9)	6.1 (12)
Skin rash	-	4 (10)	3.7 (3)	4.1 (7)
Hairloss	-	2.6 (9)	6.3 (4)	1.5 (5)
**Symptomatic at Infection**		80.3 (254)	100 (74)	74.6 (180)
**Symptom Count at Infection (0-18)**	*5.8 (4.6)*<br>*IQR=8*	*6.0 (4.6)*<br>*IQR=9*	*7.9 (3.3)*<br>*IQR=5*	*5.4 (4.7)*<br>*IQR=9*

Note

All percentages and means are weighted to be nationally representative. The unweighted numbers of observations are in parentheses

Supplementary Figure 1. Percent with Self-Reported Symptoms at Pre-Infection, Infection, and Post-Infection Stages among Those Infected Non-Long-Haulers (n=234)

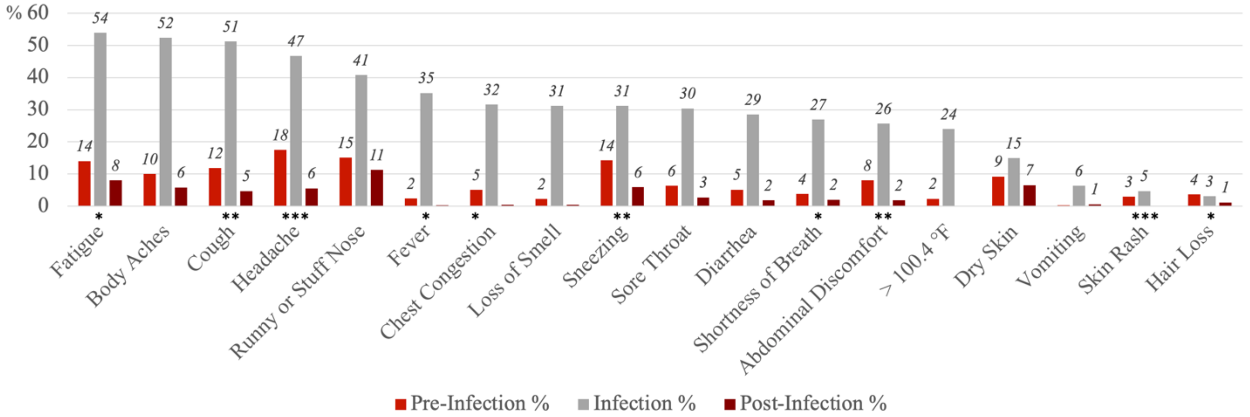

Notes

The pre-infection stage is 4 weeks before the COVID diagnosis or positive test.

The infection stage is the time of COVID diagnosis or positive test.

The post-infection stage is 12 weeks after the COVID diagnosis or positive test.

Symptoms were listed based on the proportion reported at the infection stage.

Wald (χ^2) tests were used to determine statistically significant differences in symptoms at the pre-infection stage and post-infection stage, and standard errors were clustered at the individual level.

* p<0.05 ** p<0.01 *** p<0.001
